# Evaluation of Hydrocarbon Soil Pollution Using E-Nose

**DOI:** 10.3390/s18082463

**Published:** 2018-07-30

**Authors:** Andrzej Bieganowski, Grzegorz Józefaciuk, Lidia Bandura, Łukasz Guz, Grzegorz Łagód, Wojciech Franus

**Affiliations:** 1 Institute of Agrophysics, Polish Academy of Sciences, Doświadczalna 4, 20-290 Lublin, Poland; a.bieganowski@ipan.lublin.pl (A.B.); g.jozefaciuk@ipan.lublin.pl (G.J.); 2 Faculty of Civil Engineering and Architecture, Lublin University of Technology, Nadbystrzycka 40, 20-618 Lublin, Poland; l.bandura@pollub.pl (L.B.); w.franus@pollub.pl (W.F.); 3 Faculty of Environmental Engineering, Lublin University of Technology, Nadbystrzycka 40B, 20-618 Lublin, Poland; l.guz@pollub.pl

**Keywords:** e-nose, hydrocarbon, pollution, soil

## Abstract

The possibility of detecting low levels of soil pollution by petroleum fuel using an electronic nose (e-nose) was studied. An attempt to distinguish between pollution caused by petrol and diesel oil, and its relation to the time elapsed since the pollution event was simultaneously performed. Ten arable soils, belonging to various soil groups from the World Reference Base (WRB), were investigated. The measurements were performed on soils that were moistened to field capacity, polluted separately with both hydrocarbons, and then allowed to dry slowly over a period of 180 days. The volatile fingerprints differed throughout the course of the experiment, and, by its end, they were similar to those of the unpolluted soils. Principal component analysis (PCA) and artificial neural network (ANN) analysis showed that the e-nose results could be used to detect soil contamination and distinguish between pollutants and contamination levels.

## 1. Introduction

Oil pollution, which could be toxic to humans and detrimental to environmental sensors, is now a major source of water and soil contamination due to the recent increase in extensive oil exploitation, refining, storage, distribution, and transportation [1]. A wide variety of instrumental and non-instrumental techniques are currently used to analyze oil hydrocarbons, including gas chromatography, gas chromatography–mass spectrometry, high-performance liquid chromatography (HPLC) with ultraviolet detection and mass spectrometry, size exclusion HPLC, infrared spectroscopy, supercritical fluid chromatography, thin-layer chromatography, ultraviolet and fluorescence spectroscopy, isotope ratio mass spectrometry, and gravimetric methods [2,3,4,5]. The rapid and wide-scale characterization of petroleum-contaminated soil is not feasible with traditional gas chromatography-based methods, as they are prohibitively expensive, extremely laborious, time-consuming, and they, at times, show highly variable results (an order of magnitude) across commercial laboratories, in addition to lacking field portability and warranting rigorous field sampling [6]. In recent years, emphasis was placed on the development of rapid methods and sensors to monitor different soil, water, and air contaminants. For example, a variety of methods were used to analyze hydrocarbon-contaminated soil, such as visible near-infrared reflectance spectroscopy, X-ray fluorescence, infrared spectroscopy, hyperspectral remote sensing, and Raman spectroscopy [7,8,9,10]. However, spectroscopic analysis of petrol-contaminated soils is not suitable due to volatilization losses and the significant influence of soil organic matter [11]. Detecting and monitoring these contaminants is, therefore, a challenging task [12].

Advances in multisensory material improvement technology, software innovations, and neural computation, and progress in microcircuitry design and systems integration facilitated the development of electronic noses (e-noses), which enable compounds to be discriminated by odor. The e-nose assessment systems are successfully implemented under stable laboratory conditions, for example, in the agriculture or food industry, for the prediction of rapeseed quality [13], fungal deterioration of crops [14], quality control of edible oils [15], as well as meat quality assessment [16]. The e-nose systems established a new, prospective possibility of conducting measurements in environmental monitoring [17,18,19,20,21]. Chemical sensors and e-noses are often used in odor detection or in the assessment of odor nuisance [22,23]. Capelli et al. [24] classified the application of e-noses in environmental monitoring into four main categories: air quality monitoring, water quality monitoring, process control, and pollution/odor control. They also mentioned the verification of system efficiency. Wilson [25] described various applications of e-noses to detect hydrocarbon pollution, ranging from the detection of atmospheric pollutants (gas leaks), e.g., carbon emissions from biofuel production plants and fossil-fuel production sources in the oil and gas industry, to the release of volatile organic compounds (VOCs) from numerous other industries. Most studies on petroleum and hydrocarbon pollution using e-noses concern sea water [26,27], drinking water, and wastewater contamination [28,29], in addition to industrial odors and gaseous emissions [30], as well as airborne hydrocarbons [31].

E-nose systems are not only suitable for the detection of contaminant compounds. The results obtained by Lavanya et al. support the utility of the device as a screening tool for the presence and also content of humic and fulvic acids, which are the fertility components of soil [32]. Pobkrut and Kerdcharoen proposed an interesting application of an e-nose [33]. They installed an array of six gas sensors on a semi-autonomous wheeled robot chassis in order to conduct the monitoring of soil surface for the detection of organic volatile compounds to examine the fertility. This research proved that robotized e-noses can identify and confirm the difference of soil volatiles.

Environmental applications of e-noses also include the analysis of fuel mixtures [34], the detection of oil leaks [35], and safety monitoring applications in refinery environments [36]. Ferreiro-González et al. proved that it is possible to discriminate between the petroleum-derived products poured into different surfaces [37], and to achieve post-burn sample recognition [38]. Moreover, an e-nose may be used for the quality control applications and for the fingerprinting of bitumen samples produced with crude oils of different origin [39]. This research showed that an e-nose has a number of advantages, compared to classical analytical instruments, even at room temperature.

Some research was conducted regarding the application of e-noses in the detection of soil pollutants [40,41]. The device enables the biodegradation of pollutants in soil to be monitored [42]. Attempts were also made to evaluate the soil moisture status using an e-nose [43].

E-noses can be implemented for continuous, high-frequency environmental monitoring. An online measurement system was developed and constructed to continuously monitor wastewater at the Cranfield University sewage works. High-resolution sensor profiles showed that it is possible to detect sudden changes in wastewater caused by atypical pollutants and by artificial spiking with diesel [44,45].

Despite the importance of the problem, only a few reports on the application of e-noses and online sensors for assessing hydrocarbon soil pollution can be found in the literature. Lieberman et al. [46] developed an optical-based sensor, which can be used for the direct in situ screening of subsurface petroleum hydrocarbon contaminants. The system used optical fibers to conduct remote laser-induced fluorescence measurements through a window in the probe tip. The fluorometer system directly determined the quantity of fuel products in soil in concentrations ranging from 100 to 10,000 parts per million. Apitz et al. [47] presented a system for the remote screening of petroleum hydrocarbons in soils. This system consisted of a pulsed UV laser/photodiode array fiber optic fluorometer, installed in a truck-mounted cone penetrometer. This approach enables the real-time, in situ measurement of fuel contamination and soil type to depths of 50 m. Tzing et al. [4] used two types of e-nose, with different operational principles, to identify the source of spilled oil in an accident. The suspected sources considered were petroleum oil reservoirs and pipelines located near the accident site. Subsequent principal component analysis helped identify the source. The main use of the e-nose was demonstrated to be a simple and rapid screening method to give an initial indication of the source of spilled oil. Kurup [48] described an e-nose technology integrated with an in situ vapor-sampling membrane interface probe for the rapid screening of gasoline-contaminated sites. Using a general regression neural network (GRNN), they predicted the gasoline concentration levels from the signatures obtained during the electronic nose with membrane interface probe (EN-MIP) field tests. They stated that additional field studies were needed to verify the validity of the technology in a variety of geological regimes. The abovementioned papers generally focused on the detection of polluted sites and did not conduct more detailed studies into the problem, which is considered to be rather complicated due to two main phenomena. The first is connected with the fact that petroleum products released to the environment are immediately subjected to a wide variety of weathering processes, such as evaporation, dissolution, and microbial degradation, as well as other processes such as dispersion and water–oil emulsification, photo-oxidation, adsorption onto suspended particulate materials, and oil–mineral aggregation [5]. These processes depend on the type, nature, and amount of hydrocarbons, as well as reaction time and changes to the volatile fingerprints of the contaminated soils. The second problem is related to changes in soil moisture, as indicated by Bourgeois et al. [49]. They stated that a careful system design and sample preconditioning can help minimize changes in the relative humidity of the sample. Considering the practical application of a sensor array, this can make the overall instrument more complex and expensive, and can also affect its portability or limit sample throughput. The alternative approach is to measure these parameters and calibrate the sensors under varying humidity levels in order to compensate for changes in subsequent data analysis. This parametric compensation approach was favored in a number of applications where relative humidity (RH) was used as an input to artificial neural networks.

In the present paper, we tried filling these gaps by studying artificially polluted soils coming from different geological sites a long time after they were polluted, to allow for the development of oil degradation and changes in soil humidity. We used two different pollution agents, commercial gasoline and diesel oil, to differentiate between various kinds of hydrocarbons. The volatile fingerprints of the soil registered by the e-nose were interpreted using principal component analysis (PCA) and artificial neural networks (ANN).

## 2. Materials and Methods

*Soils*

Ten different mineral soils sampled from the arable layer (at a depth of 5–20 cm) were used. Their basic properties are summarized in Table 1.

*Petroleum Contaminants*

Two commercial fuels, EuroSuper 98 petrol and Ekodiesel, purchased from PKN Orlen (a Polish petroleum company) were used in the experiment. The characteristics of both fuels provided by the producer are as follows: EuroSuper 98 is a mixture of low-boiling hydrocarbons, organic oxygen compounds, and enriching additives (below 1%), including detergents, anticorrosion, and antioxidant additives. It contains up to 35% *v*/*v* aromatic hydrocarbons, 18% *v*/*v* unsaturated hydrocarbons, 1% *v*/*v* benzene, 3% *v*/*v* methanol, and 5% *v*/*v* ethanol. EuroSuper 98 is a clear and transparent liquid with a density ranging between 0.720 and 0.775 g/cm^3^, and a viscosity of around 0.6 mm^2^/s. Ekodiesel is a mixture of C9–C25 hydrocarbons, fatty-acid methyl esters, and enriching additives. It contains up to 7% *m*/*m* of polycyclic aromatic hydrocarbons. It is a yellow-colored liquid with a density of 0.82–0.845 g/cm^3^ (15 °C), and a viscosity of 2.0–4.5 mm^2^/s. Additional chromatographic analysis performed using the Trace GC Ultra gas chromatograph, coupled with the PolarisQ (Thermo) mass spectroscope (Thermo Electron Corporation, Austin, TX USA), revealed that both fuels contain mainly aliphatic hydrocarbons. EuroSuper 98 also contains a certain amount of benzene derivatives (toluene, ethylbenzene, chlorobenzene, propylbenzene, and xylenes), whereas fatty-acid methyl esters and benzene derivatives (tri- and tetramethylbenzene, and dimethylbenzene) were identified in Ekodiesel.

*Experimental Set-up*

**Figure 1:**
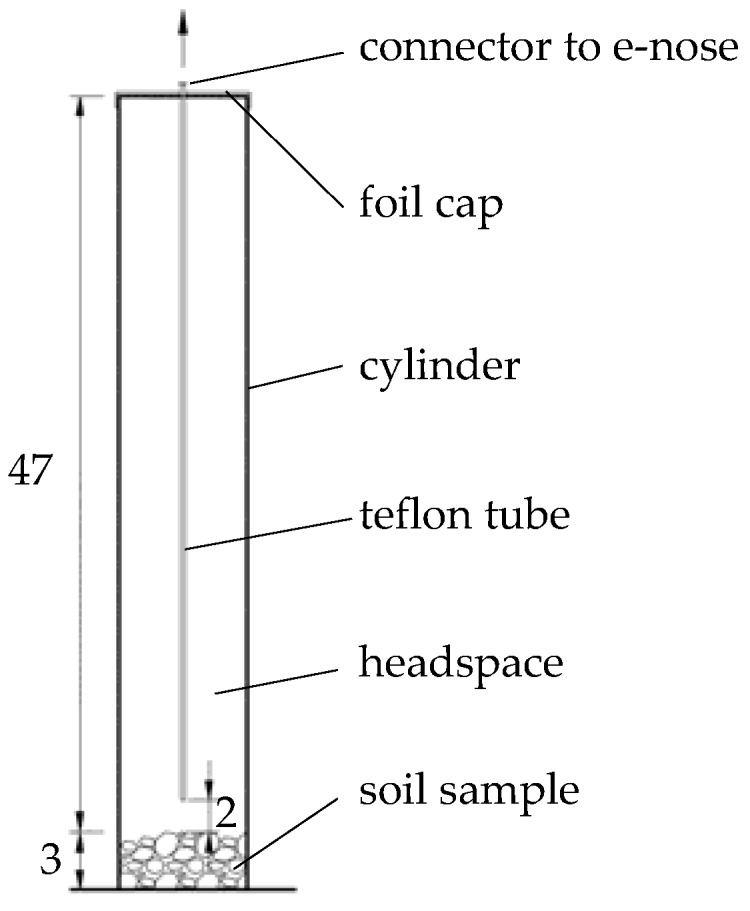
Schema of sampling method (dimensions in cm).

The soils were stored for around a year in air-dried conditions before being gently crushed in a mortar and put through a 2-mm sieve. The soil samples (75 g) were moistened with 15 mL of water, placed in 2.5-dm^3^ glass cylinders that were carefully washed and dried at 200 °C, and covered with a sterile aluminum foil with a 3-mm hole (diameter) in its center. This meant that the soil could be slowly dried and most of the soil emissions could be maintained in the cylinder headspace (Figure 1).

Over the whole experimental cycle, the cylinders were stored in a dark chamber ventilated with synthetic air at a temperature of 20 ± 1 °C. In order to ensure that all cylinders were the same (in the context of experiments), the e-nose signals were measured in each empty cylinder. The results from this series of measurements were stable and the same for all cylinders. The next step was to check whether our e-nose was able to distinguish the gas fingerprint of petrol and diesel. We added the same amount (as later to the soils) of both petroleum products to two empty cylinders for this purpose. The analysis of the e-nose readout from the clean cylinder, polluted with petrol and polluted with diesel, revealed that all three objects differed significantly.

Before each measurement of contaminated soil, the e-nose sensors were flushed with synthetic air for 10 min, and an additional two minutes elapsed before each soil sample was measured again. For the measurement of soil volatile fingerprints, a polyamide tube (with an inner diameter of 2 mm) connected with an e-nose sensing chamber was introduced through the aluminum-foil hole into the cylinder. The inlet to the tube was established 2 cm above the surface of the soil. The membrane micro-pump (FM1101 F6V Fürgut GmbH) sucked the air out from a chamber at a speed of 100 cm^3^ min^−1^. Such suction sampled only about 20% of the cylinder volume during five minutes of the measurement. It enabled minimizing the gas exchange between the cylinder and atmosphere. The measurement was conveyed with about 1-Hz reading frequency. The average value from 45 e-nose instrumental readings was taken to PCA analysis from the most stable region at the end of the measurements. The ratios (relative resistances) were further calculated for the soil headspace air and for the synthetic air. It enabled obtaining 744 records. This value was obtained from the following calculation: we had 10 soil types with three variants of soil contamination (i.e., one control, one with petrol, and one with diesel). For each session, we also measured an empty (blank) cylinder. Performing three replicates allowed obtaining 93 measurements in one cycle. Taking into account the eight days (cycles) in which the measurements were carried out, the total number of records was 744. In the case of ANN learning, a small set of data was used. In order to increase the volume of the training set, about 13 values from each measurement were selected with an interval of approximately 3 s from the same time interval. This allowed 9656 rows of data to be obtained.

*Experiment*

Three series of all soil samples were prepared and placed in the cylinders. One of them was polluted with 1 µL of petrol, the second with 1 µL of diesel, and the third (control) remained unpolluted. The e-nose signals were registered after 1, 8, 15, 37, 64, 93, and 173 days. Prior to each measurement, the soil moisture was determined by weighing the entire soil-filled cylinder. The relative humidity (RH) and temperature (T) of the cylinder atmospheres were measured using the Honeywell HIH-4000 and Dallas DS18B20 sensors, respectively. Relative humidity was monitored during the whole experiment, and was stable (at the level ~50%). Each experiment was replicated three times.

*E-Nose*

The e-nose device used was constructed from eight relatively small, metal-oxide semiconductors (MOS) with a low power consumption (ca. 300 mW), manufactured by TGS Figaro: 1—TGS2600-B00, 2—TGS2610-C00, 3—TGS2611-C00, 4—TGS2612-D00, 5—TGS2611-E00, 6—TGS2620-C00, 7—TGS2602-B00, and 8—TGS2610-D00. Changes in the electric conductivity of the sensing elements, due to surface chemical reactions between gas molecules and the semiconductor, provide a signal response depending on the composition and concentration of the gas. The signal is different for each sensor. The MOS sensors applied in the measuring device were distributed in a circular array, covered with a head providing an equal flow of gas and a stable temperature in the measurement chamber. Before the experiment, the sensors were pre-calibrated with a set of single chemical substances of standard concentrations, specific to each particular sensor. The signals from all eight implemented sensors constituted a full e-nose array response. A detailed description of the equipment was presented by Guz et al. [50]. 

*Data Analysis*

PCA [51,52] and ANN [53,54,55] methods were used to interpret the data obtained. 

The PCA analysis enabled the selection of new independent variables (described here on the axes as PC1 and PC2) best describing the variability of the analyzed dataset. The new designed variables had no direct physical meaning and showed their percentage contribution to the total covariance of the dataset.

The ANN was applied for two purposes:

(1) To find similarities and differences between the ANN classification of the samples using pattern recognition. Here, the architecture of the 10 networks used consisted of eight inputs, one hidden layer with 16 neurons, and two or three output neurons according to the number of target output classes. Training of the networks was performed using a scaled conjugate gradient backpropagation algorithm, and the error was estimated using cross entropy (CE).
(2) ANN (two networks) function approximation and nonlinear regression were used to estimate the time lapse from when pollution was initiated. The network architecture consisted of eight inputs, one hidden layer with 16 neurons, and one output neuron. Five different networks were used in the case of petrol and ecodiesel pollutants. Training of the networks was performed using the Levenberg–Marquardt algorithm and the error was estimated using the mean squared error (MSE).

The learning (70%), testing (15%), and validation (15%) data subsets were chosen randomly from the entire dataset. The training data were presented to the networks during training, and the networks were adjusted according to their error. Validation was used to measure network generalization and to finish training when generalization stopped improving. Testing the data had no effect on training, and thus, provided an independent measure of network performance during and after training.

## 3. Results and Discussion

**Figure 2:**
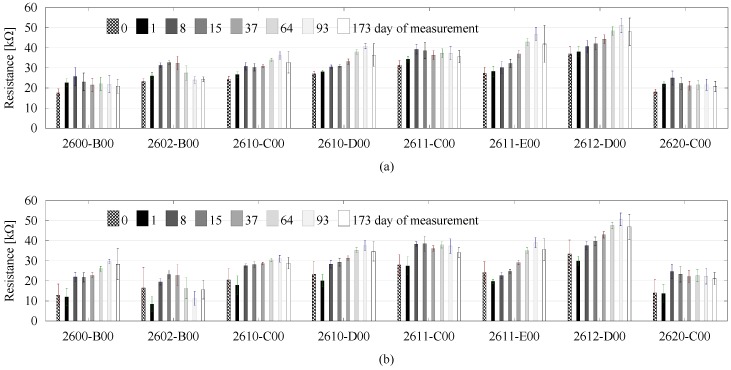

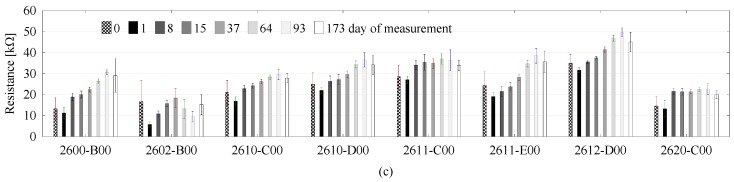
Raw signal changes of electronic nose (e-nose) particular sensors during experiment (**a**) without contamination, (**b**) contamination with diesel, and (**c**) contamination with petrol. Error bars represent standard deviation.

The average value of resistance (kΩ) from the last 45 s of measurements for particular gas sensors are presented in Figure 2. Generally, the value of signal in the electrical circuit of these sensors is inversely proportional to the concentration of volatile compounds and pollutants. It is noticeable that, during conducted measurements, it shifted constantly for the non-modified samples. The soil samples were slowly dried out naturally since the beginning of the measurements. According to Lavanya et al. [32], the moist soil samples are characterized by a more intensive smell profile than the dry samples. After spitting fuel into the soil samples, the concentration of pollutant fumes in the gaseous phase increased, which was found during the subsequent measurement session. The return of signal responses to the incipient values was observed in the second day after contamination for the following gas sensors: 2600-B00, 2610-D00, 2611-C00, and 2620-C00. For the other sensors, this fact was observed on the 15th day (2612-D00), 37th day (2602-B00), and 64th day (2611-E00). Alignment of the sensor response to the incipient value does not unequivocally mean that all doses of fuel were already evaporated. The fingerprint differences between the blank and contaminated soil samples were still noticeable.

**Figure 3:**
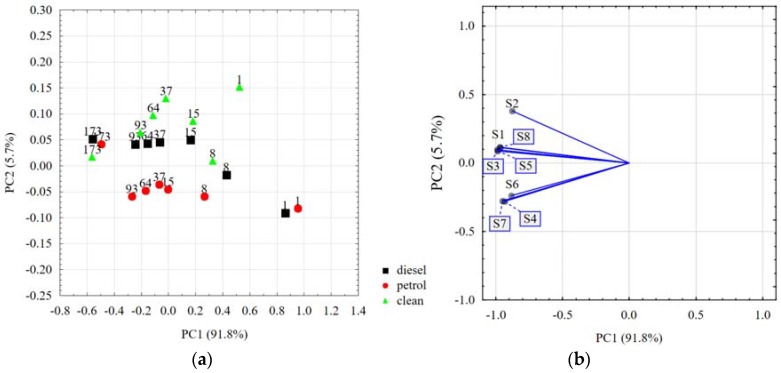
Two-dimensional principal component analysis (PCA) plots for (**a**) the volatile fingerprints of the 10 soils under examination, polluted with petrol and diesel. The numbers represent days after pollution. The points represent the averaged data for all ten soils. (**b**) Plot of variable loadings.

Results of the PCA analysis pertaining to the volatile fingerprints of the soils under examination are presented in Figure 3a. Using PCA, we reduced the eight-dimensional data space (eight e-nose sensors) to a two-dimensional data covariance matrix, with the x-axis representing 91.8% and the y-axis 5.7% of the whole covariance. This method only lost 2.5% of the information, which is a very good level. This is a certain degree of simplification; however, it enables presenting the results using standard two-dimensional charts. The results of variable loadings (particular sensors) are presented in Figure 3b. The responses of all sensors were similar in direction. All sensors were closely related to the first principal component.

One day after pollution, the PCA results of the petrol- and diesel-contaminated soils were located in the nearby neighborhood, far away from the unpolluted control soils. It is highly probable that, in the short term (hours to days) after a spill, evaporation is the single most important and dominant process, particularly in terms of light petroleum products, affecting the volatile fingerprints to the highest extent. In the first few days following a spill, the loss in volume of light crudes and petroleum products can be up to 70% and 40%, respectively. The loss in volume of heavy or residual diesels is only about 5 to 10% [5]. In the period of eight to 93 days after pollution, the PCA results of petrol- and diesel-contaminated soils were located on separate lines of differing locations, mainly against the PC2 axis, which indicates differences in the fingerprints of both pollutants. Sobanski et al. [56] proved there to be various fingerprints for different fuels, using an e-nose system to satisfactorily classify different types of petrol, heating oil and diesel oil, using a gas measurement system based on a thick-film semiconductor sensor matrix. Similarly, Brudzewski et al. [57] used the e-nose measurement system, together with a support vector machine (SVM), to classify the gasoline with supplement of bioproducts, showing that the semiconductor sensors array responds with a unique signal pattern for each petrol blend type, and can be used to recognize different types of petrol blend. Over the period of eight to 93 days, the points for unpolluted soils were located above those of the polluted soils, exhibiting the highest dispersion, and they were sometimes close to the diesel-contaminated objects. A dispersion of the points for unpolluted soils may be connected with the activity of biological life, which is not retarded by toxic compounds. Different volatile substances produced by living microorganisms, including the products of soil organic matter decay, evolve depending on the length of time taken for the soil moistening to alter the e-nose response. Full microbial activity starts between two to seven days after dry soil is moistened [58], and this could shift the PCA results down and leftwards, and then right and upwards, causing a large dispersion of the e-nose signals observed from the eighth to 37th day of our experiments. Subsequently, water evaporation (replacement of water by air in some pores, with the water still present in the smaller ones) allowed aerobic bacteria to develop, causing changes in the ratio of different microorganisms, and, along with the suppression of all biological life at the course of water evaporation, this may change the further course of the observed PCA results (left and downwards). On the last, or 173rd, day of the experiment, the PCA points for all soils met at close positions, regardless of whether or not the soils were polluted. By this time, in air-dried soil, the microbial populations reached a dormant state, and the pollutants were possibly evaporated to a small, hardly detectable level.

Since the PCA analysis differentiated the volatile fingerprints of all of the soils at various time points during the experiment, we checked whether these differences were significant enough to allow the neural networks to distinguish between the polluted and unpolluted soils, as well as the kind of pollution. 

At first, we tried distinguishing whether or not the soil was polluted, along with the kind of pollutant, using a single-step classification for 9656 data rows according to the following scheme: 
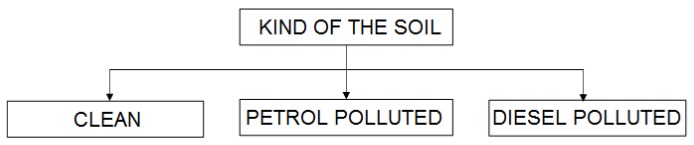


Table 2 shows the results of the classification. Trying to minimize errors of the abovementioned single-step classification, we decided to perform a two-step procedure involving an initial distinguishing between polluted and unpolluted soils, before further distinguishing the kind of pollutant, according to the following scheme:
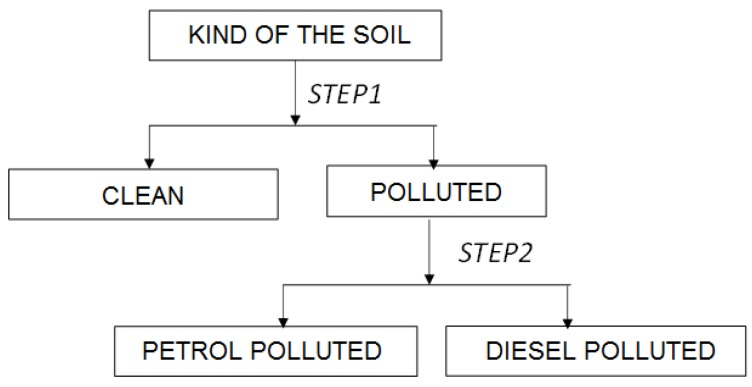


The first step was performed for all 9656 data rows, and the second for 6423 data rows containing the soils classified as being polluted. The data were not subjected to dimensional reduction as in the case of PCA analysis.

Table 3 shows the results of the two-step classification presented above. The two-step classification results were quite satisfactory, approaching a near-100% certainty that the volatile fingerprints of soils, measured at various time points, could distinguish between the clean and polluted soils, as well as being able to identify the kind of pollutant. 

Additionally, the analysis was conducted with ANNs for one chosen type of soil, the measurement data of which were not used for network learning (see Table 4). The task of the network was to determine whether a given sample was contaminated or not. The analysis was carried out in two variants: when the time after pollution was known, and when no such information was available. In the first case, the prediction errors fell within the range of 7.4 to 16.6%. The smallest error was obtained on the first day after the addition of pollutants. However, when the period from pollution was not known, the average prediction error was much larger, and amounted to 21.3% on average.

**Figure 4:**
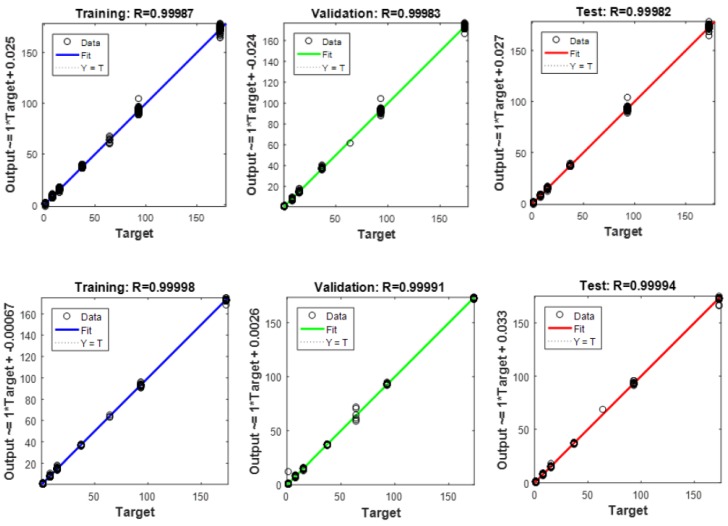
Exemplary fits of artificial neural network (ANN) identifier (ID) 1 for the predicted and measured data for the estimation of the time lapse from the beginning of pollution with petrol (**up**) and diesel (**down**).

The results of the neural networks’ ability to find a function able to distinguish data samples collected at different pre-defined days after pollution are presented in Table 5. The results suggest that the e-nose could be used for assessment of the time lapse from the beginning of the soil being polluted with 1 µL of petrol and 1 µL of diesel. The results also show a very good agreement between the expected and measured data presented in Figure 4. 

The results presented above indicate that the concentration of the pollutants, as related to the time of their evaporation, could also be detected.

## 4. Conclusions

The research described in this work presents the possibility of applying e-nose sensors to the evaluation of hydrocarbon pollution and its extent for several soil types. Data post-processing, using the PCA method and ANNs, enabled us to distinguish between the clean and polluted soils, as well as to estimate the time elapsed since the beginning of the pollution.

The PCA plot showed that volatile fingerprints of clean soils varied to the greatest extent, something we attributed to the diversity of bacterial populations in various soils, as well as to a variety of volatile substances produced by soil microorganisms.

The differences in volatile fingerprints detected using PCA analysis were encouraging for the ability of ANN to recognize soil polluted with petrol and diesel, as well as its status, despite the differences between soil type and origin. The neural networks applied gave a validation quality of over 95% (Table 2), proving that there were very few mistakes in the classification of the validation set. This result is a very good starting point for the use of the e-nose/ANN combination in further investigations of soil pollution.

The combination of e-nose volatile fingerprint measurements, PCA analysis for the enhancement of differences, and artificial neural networks for classification forms a suitable tool for recognizing hydrocarbon soil pollution. The effectiveness of classifying a sample as being polluted with petrol and/or diesel can reach a level of almost 100%.

## Figures and Tables

**Table 1:** Basic properties of the investigated soils.

No.	WRB Soil Group	C_org_ (%)	Particle Size Group
1	Brunic Arenosol	0.86	Sand
2	Stagnic Luvisol	1.19	Sandy loam
3	Haplic Cambisol	0.57	Sandy loam
4	Leptic Cambisol	1.08	Silt loam
5	Mollic Stagnic Fluvisol	1.14	Silt loam
6	Stagnic Phaeozem (Siltic)	1.97	Silt
7	Haplic Chernozem (Siltic)	1.11	Silt loam
8	Haplic Luvisol (Siltic)	1.06	Silt
9	Leptic Skeletic Dystric Cambisol	0.90	Silt loam
10	Haplic Fluvisol (Clayic)	1.86	Silt

**Table 2:** Classification errors for the networks used, using a single-step classification of all the soils studied.

%E *			
Network ID	Training	Validation	Testing
1	17.44	15.98	17.40
2	18.83	18.95	19.82
3	17.20	17.09	17.50
4	17.32	16.98	18.64
5	18.11	19.47	20.44
6	18.00	18.33	17.57
7	17.85	17.78	17.43
8	17.22	17.99	18.09
9	17.92	16.71	18.26
10	17.17	17.18	17.67
**Average**	**17.71**	**17.65**	**18.28**

* Percentage error (%E) indicates the fraction of samples which were misclassified. A value of 0 means no misclassifications, while a value of 100 indicates the maximum number of misclassifications. ID—identifier.

**Table 3:** Classification errors for the networks used in the in two-step classification of all the soils studied.

STEP1				STEP2			
Network ID	%E *			Network ID	%E *		
	Training	Validation	Testing		Training	Validation	Testing
1	0.90	0.62	0.96	1	3.98	4.15	3.53
2	6.46	5.83	7.32	2	3.98	4.25	3.94
3	0.76	0.41	1.03	3	4.38	3.42	3.32
4	4.03	4.07	3.86	4	3.75	3.63	4.15
5	6.86	6.35	8.14	5	3.80	4.46	3.01
6	5.63	5.17	5.73	6	3.46	3.42	3.32
7	1.92	1.17	2.48	7	4.88	5.28	3.94
8	1.06	1.45	1.38	8	4.04	3.21	3.94
9	1.71	1.65	2.27	9	4.51	4.36	3.53
10	3.59	3.31	4.07	10	3.82	4.88	3.63
**Average**	**3.29**	**3.00**	**3.72**	**Average**	**4.06**	**4.11**	**3.63**

* Percentage error (%E) indicates the fraction of samples which were misclassified. A value of 0 means no misclassifications, while a value of 100 indicates the maximum number of misclassifications.

**Table 4:** Classification errors for the artificial neural networks (ANNs) used in the prediction of one chosen type of soil contamination assessment (yes/no).

ANN ID	Day Known							Day Unknown
	1	8	15	37	64	93	173	1–173
1	7.2	8.9	10.8	12.0	10.6	13.6	15.6	21.7
2	6.1	11.1	9.9	8.4	13.8	19.1	17.4	20.7
3	8.3	14.4	14.8	14.4	14.2	14.0	13.9	23.7
4	7.6	11.9	12.2	13.3	13.3	13.3	12.8	15.4
5	6.1	9.8	14.1	9.2	14.5	19.4	15.0	19.1
6	6.3	8.8	10.7	12.0	13.8	18.6	17.2	24.7
7	8.3	10.7	12.2	14.6	12.9	17.9	15.0	24.4
8	7.4	8.8	14.7	8.4	14.3	15.5	12.2	18.0
9	7.7	8.7	8.2	8.0	13.3	17.7	12.2	23.3
10	9.7	8.2	9.2	13.3	11.6	16.4	14.8	21.5
**Average**	**7.5**	**10.1**	**11.7**	**11.4**	**13.2**	**16.6**	**14.6**	**21.3**

**Table 5:** Efficiency of the used networks in distinguishing the data samples collected at different pre-defined days after pollution.

**Petrol**						
**Network ID**	**Training**		**Validation**		**Testing**	
	**MSE**	**R**	**MSE**	**R**	**MSE**	**R**
1	0.99	0.99	1.14	0.99	1.27	0.99
2	0.06	0.99	0.19	0.99	0.08	0.99
3	0.16	0.99	0.18	0.99	0.48	0.99
4	0.032	0.99	0.013	0.99	0.023	0.99
5	0.10	0.99	0.26	0.99	15.58	0.97
**average**	**0.27**	**0.99**	**0.36**	**0.99**	**3.49**	**0.99**
**Diesel**						
**Network ID**	**Training**		**Validation**		**Testing**	
	MSE *	R *	**MSE**	**R**	**MSE**	**R**
1	0.11	0.99	0.69	0.99	0.40	0.99
2	0.29	0.99	0.56	0.99	0.71	0.99
3	0.10	0.99	0.21	0.99	0.90	0.99
4	0.29	0.99	0.30	0.99	0.93	0.99
5	1.33	0.99	1.27	0.99	1.65	0.99
**average**	**0.42**	**0.99**	**0.61**	**0.99**	**0.92**	**0.99**

* The mean squared error (MSE) is the average squared difference between outputs and targets. Lower values are better, whereby a value of 0 means no error. Regression (R) values measure the correlation between outputs and targets. An R value of 1 means a close relationship, while a value of 0 corresponds to a random relationship.
